# Distinct Ubiquitin Binding Modes Exhibited by SH3 Domains: Molecular Determinants and Functional Implications

**DOI:** 10.1371/journal.pone.0073018

**Published:** 2013-09-11

**Authors:** Jose L. Ortega Roldan, Salvador Casares, Malene Ringkjøbing Jensen, Nayra Cárdenes, Jerónimo Bravo, Martin Blackledge, Ana I. Azuaga, Nico A. J. van Nuland

**Affiliations:** 1 Departamento de Química Física e Instituto de Biotecnología, Facultad de Ciencias, Universidad de Granada, Granada, Spain; 2 Department of Biochemistry, University of Oxford, Oxford, United Kingdom; 3 Protein Dynamics and Flexibility by NMR, Institut de Biologie Structurale Jean-Pierre Ebel, CEA, CNRS, UJF UMR 5075, Grenoble, France; 4 Vascular Medicine Institute, University of Pittsburgh, Pittsburgh, Pennsylvania, United States of America; 5 Instituto de Biomedicina de Valencia, IBV-CSIC, Valencia, Spain; 6 Structural Biology Brussels, Vrije Universiteit Brussel (VUB), Brussels, Belgium; 7 Department of Structural Biology, VIB, Brussels, Belgium; Hungarian Academy of Sciences, Hungary

## Abstract

SH3 domains constitute a new type of ubiquitin-binding domains. We previously showed that the third SH3 domain (SH3-C) of CD2AP binds ubiquitin in an alternative orientation. We have determined the structure of the complex between first CD2AP SH3 domain and ubiquitin and performed a structural and mutational analysis to decipher the determinants of the SH3-C binding mode to ubiquitin. We found that the Phe-to-Tyr mutation in CD2AP and in the homologous CIN85 SH3-C domain does not abrogate ubiquitin binding, in contrast to previous hypothesis and our findings for the first two CD2AP SH3 domains. The similar alternative binding mode of the SH3-C domains of these related adaptor proteins is characterised by a higher affinity to C-terminal extended ubiquitin molecules. We conclude that CD2AP/CIN85 SH3-C domain interaction with ubiquitin constitutes a new ubiquitin-binding mode involved in a different cellular function and thus changes the previously established mechanism of EGF-dependent CD2AP/CIN85 mono-ubiquitination.

## Introduction

Adaptor molecules are non-catalytic polypeptides that contain one or more domains able to bind to other proteins or non-protein ligands [1]. These molecules selectively control the spatial and temporal assembly of multi-protein complexes that transmit intracellular signals involved in regulation of cell growth, differentiation, migration and survival. The importance of such signalling networks is well understood for signal transduction induced by receptor tyrosine kinases (RTKs) [2].

Down regulation of RTKs is a critical step in modulating their activity [3]. This is a highly ordered and dynamic process controlled by the assembly of a large network of RTK-associated proteins as well as their post-translational modifications including phosphorylation and ubiquitination. The Cbl family of ubiquitin ligases plays a major role in receptor ubiquitination, receptor sorting for degradation and ultimately in cessation of receptor-induced signal transduction. Several lines of evidence support a role of CMS/CIN85 (Cas ligand with Multiple SH3 domains/Cbl-Interacting protein of 85 kDa) in the regulation of Cbl-directed RTK down regulation [4–7]. CMS, also known as CD2AP (CD2 associated protein), shares overall domain organization with CIN85, containing three SH3 (Src-Homology) domains, a proline-rich region and a coiled-coiled domain, and high sequence identity and hence, it has been assumed to belong to the same family of ubiquitously expressed adaptor molecules and elicit similar biological functions. The three N-terminal SH3 domains, named A, B and C, share higher similarity among themselves than to any other SH3 domain, suggesting that they may have overlapping specificities in binding [8].

SH3 domains form a highly conserved family of protein domains, but their amino acid composition varies at a few key sites, allowing for a wide range of molecular targets. Similar to SH3 domains, ubiquitin-binding domains (UBDs) are found in proteins with different biological function. Structures of many complexes between UBDs and ubiquitin have been determined either by X-ray crystallography or high-resolution NMR (reviewed in 9,10). Recently, SH3 domains were identified as a new class of UBDs [11]. Since then, several low- and high-resolution structural studies have raised a significant number of unanswered questions concerning the determinants of the specificity of the interaction between SH3 domains and ubiquitin [12–15]. Moreover, several mechanistic differences have been proposed for ubiquitin binding to SH3 domains involved in the immune signalling pathway [16].

A key affinity and specificity determinant has been appointed to Phe409 in Sla1 SH3-3 and the three SH3 domains of CIN85. We recently used NMR residual dipolar couplings (RDCs) to obtain the solution structure of the complex between ubiquitin and the third (SH3-C) domain of CD2AP [15], and showed that this SH3 domain binds ubiquitin in an alternative orientation.

In the current work, we have performed structural and mutational analysis of the three SH3 domains of CD2AP and the third SH3 domain of CIN85. We have found a distinct ubiquitin-binding mode for the third SH3 domains of both CD2AP and CIN85 adaptor proteins, in which i. the Phe corresponding to Phe409 in Sla1 SH3-3 does not play a key role, and *ii*. Is characterized by a higher affinity for ubiquitin that is augmented by additional stretches of residues at the C-terminus of ubiquitin. The differences in binding mode of SH3 domains to ubiquitin might be related to different functions inside the cell. We propose that the CD2AP/CIN85 SH3-C interaction with ubiquitin is related to selective binding to ubiquitin molecules covalently bonded via its C-terminus in ubiquitinated CD2AP and CIN85.

## Materials and Methods

### Protein expression and purification

Unlabelled, ^15^N-labelled and ^13^C,^15^N CD2AP SH3-A and C were obtained as described elsewhere [17]. Unlabelled CD2AP SH3-B was expressed as described previously [17]. Unlabelled and ^15^N-labelled His-tagged and non His-tagged ubiquitin were purchased from Spectra Isotopes or obtained as described in [12] [18]. Protein concentrations were determined by UV-absorption measurements at 280 nm using extinction coefficients of 9970, 12660, 13980, 13980 and 1450 M^-1^ cm^-1^ for CD2AP SH3-A, B and C, CIN85 SH3-C and ubiquitin respectively, determined using the ProtParam algorithm (www.expasy.ch).

The genes encoding the mutants CD2AP F324Y SH3-C and CIN85 F322Y SH3-C proteins were obtained by site-directed mutagenesis using QuikChange^TM^ (Stratagene) kit. Mutant proteins were over-expressed in *E. Coli* BL21/DE3 cells and purified like the WT CD2AP SH3-C and WT CIN85 SH3-C domains. F53Y CD2AP SH3-A mutant and CD2AP SH3-C T283A and E302K + T303 deletion mutants were purchased from Topgene and expressed as the WT domains. Both the DNA sequence and molecular weight of the final purified protein were checked to confirm the presence of every mutation.

The CIN85-SH3C _270-328_His_6_ was cloned in pET21a and expressed in *E. coli* BL21 (DE3) grown in LB broth after 7 hours induction with 0.1 mM IPTG at 25^°^C. Cell pellets were lysed by sonication with 50mM Tris pH 8.0, 200mM NaCl and 50mM Imidazole. After sonication, the lysate was subjected to His-tag selection in a nickel charged HiTrap chelating column. The protein was eluted in a 50-500 mM imidazole gradient. After chelating the sample was diluted up to 20mM imidazole and loaded into a Q FF HiTrap column (Ge Healthcare), washed with 20mM Hepes pH 8.6 and eluted in a NaCl gradient. Finally a size exclusion on a Superdex75 column using 20mM Hepes pH 8.0, 100mM Nacl and 4% glycerol was performed.

### NMR chemical shift perturbation

All NMR titration experiments were recorded at 25°C on a Varian NMR Direct-Drive 600 MHz spectrometer (^1^H frequency of 600.25 MHz) equipped with a triple-resonance PFG-XYZ probe. ^15^N labelled-CD2AP SH3-A and C samples and ubiquitin were prepared in 92% H_2_O/8% D_2_O, 50 mM NaPi, 1 mM DTT at pH 6.0. A HNCACB triple resonance spectrum was recorded on a ^13^C,^15^N-labelled ubiquitin to confirm the backbone assignment at pH 6.0. CD2AP SH3-A and C backbone resonances were previously assigned at pH 6.0 (BMRB accession numbers 16641 and 16643).

The ubiquitin-binding site on all SH3 domains site was obtained by titrating with increasing amounts of unlabelled ubiquitin into a 0.2 mM ^15^N-labelled SH3 sample. The SH3-binding site on ubiquitin was obtained by titrating with increasing amounts of unlabelled SH3 domain into a 0.25 mM ^15^N-ubiquitin sample. The progress of the titrations was monitored by recording one-dimensional ^1^H and two-dimensional ^1^H-^15^N HSQC spectra. The magnitude of the chemical shift deviations (Δδ) was calculated using the equation:

$$\Delta \delta =\sqrt{{\left(\Delta \delta_{HN}\right)}^{2}+{\left(\frac{\Delta \delta_{N}}{6.51}\right)}^{2}}$$

where the difference in chemical shift is that between the resonances corresponding to the bound and free forms at each titration point.

All NMR data were processed using NMRPipe [19] and analyzed by NMRView [20] and Sparky [21].

### Measurement of RDCs and structure calculation

Partially aligned samples in a liquid-crystalline medium consisting of a 5% penta-ethyleneglycol monododecyl ether (C_12_E_5_)/hexanol mixture [22] were prepared for RDC measurement. RDCs were measured for the CD2AP SH3-C:Ubiquitin and CD2AP SH3-A:Ubiquitin complexes as described elsewhere [15].

The structure calculation of the CD2AP SH3-A and C domains in complex with ubiquitin were carried out using the program SCULPTOR [23] as described previously [15]. The ten lowest target function structures (combining RDC and AIR violation for the CD2AP SH3-A complex and RDC, AIR and nOe violation for the CD2AP SH3-C complex) were used for the final analysis. Validation of the structural quality was done using PROCHECK [24,25]. H-bonds and non-bonded interactions were obtained from analysis done with the LIGPLOT software [26]. Salt-bridges and buried surface areas were obtained from analysis using the EBI PISA web server [27].

### Crystallization, Crystallographic Data Collection, Structure Determination, and Refinement of CIN85 SH3-C

The protein was concentrated at 11.4 mM and crystallized at 4^°^C by vapor diffusion method using 1.8M ammonium sulphate, 0.1M Citrate pH 6.0. Crystals were stepped soaked prior to crystal diffraction until 15-20% glycerol.

Data reduction was undertaken with Mosflm and Scala [28], Table **S2**). Crystals belong to the P3 _1_21 space group and were solved by molecular replacement using the Sem5 C-terminal SH3 (PDB ID: 1sem) using the program Molrep [28]. Automated model building was carried out with the program ARP/wARP [28]. Cycles of manual and automatic refinement were performed with Coot [29] and REFMAC [28] respectively. The final model includes residues 270-328 from CIN85 plus 2 and 6 additional residues at the N and C terminus respectively. All residues are in the favored regions of the Ramachandran plot.

### Fluorescence binding experiments and Isothermal Titration Calorimetry (ITC)

Prior to the ITC experiments, fluorescence measurements were made on a Varian, Cary Eclipse spectrofluorimeter equipped with a peltier temperature-controlled cell holder. Intrinsic fluorescence emission spectra were recorded at 25^°^C, from 305 to 400 nm with an excitation wavelength of 298 nm to predominantly excite the tryptophan residues present only in the SH3 domain. Each spectrum was the result of 5 accumulations collected at a scan rate of 200 nm·min^-1^. First, the spectra were recorded in the absence of ubiquitin for CD2AP SH3-C WT (30 µM) and its mutant F324Y (40 µM) and CD2AP SH3-A (20 µM) samples. Each protein sample was titrated by addition of increasing volumes of a concentrated ubiquitin stock solution (760µM) up to a final SH3: Ubiquitin molar ratio of ~1:20. Emission fluorescence spectra were recorded for each titration point. All spectra were blank-corrected and normalized by protein concentration. In order to estimate apparent affinities, corrected emission spectra were integrated between 310 (to exclude signal scattering) and 400 nm to get the total emitted fluorescence (quantum yield), which was then finally fitted to a model of a single set of identical sites.

ITC experiments were performed at 25°C in a high-precision MCS isothermal titration calorimeter and in a VP isothermal titration calorimeter (Microcal Inc., Northampton, USA). Prior to the experiments, WT and F324Y CD2AP SH3-C, WT CD2AP SH3-A, as well as ubiquitin were extensively dialyzed at 4°C against the appropriate buffer (20 mM Cacodylate, pH 6.0). All buffers and solutions were filtered, degassed and equilibrated to the working temperature prior to each experiment. Typically, a 50 µM CD2AP SH3 solution in the calorimeter’s cell was titrated with the ubiquitin stock solution (at a concentration of about 800 µM). Due to the relatively low binding affinities, titrations were made by using a profile of injection volumes of the ubiquitin stock solution in order to define the titration isotherm curve more accurately. As a blank, an independent experiment with only buffer in the calorimeter’s cell was performed with the same ubiquitin stock solution to determine the corresponding heats of dilution. The dilution isotherm was subtracted from that obtained for the titration of the protein. The area under each peak of the net thermogram was integrated to determine the heat produced by each binding event between the ubiquitin and the SH3 domain after each injection.

The binding constant, K_b_, and the enthalpy change of binding, ΔH_b_, were determined by fitting of the binding isotherms, using a model of binding to a single set of identical sites as described previously [30].

## Results

### NMR titration experiments of CD2AP SH3-A and C domains with ubiquitin

**Figure 1:**
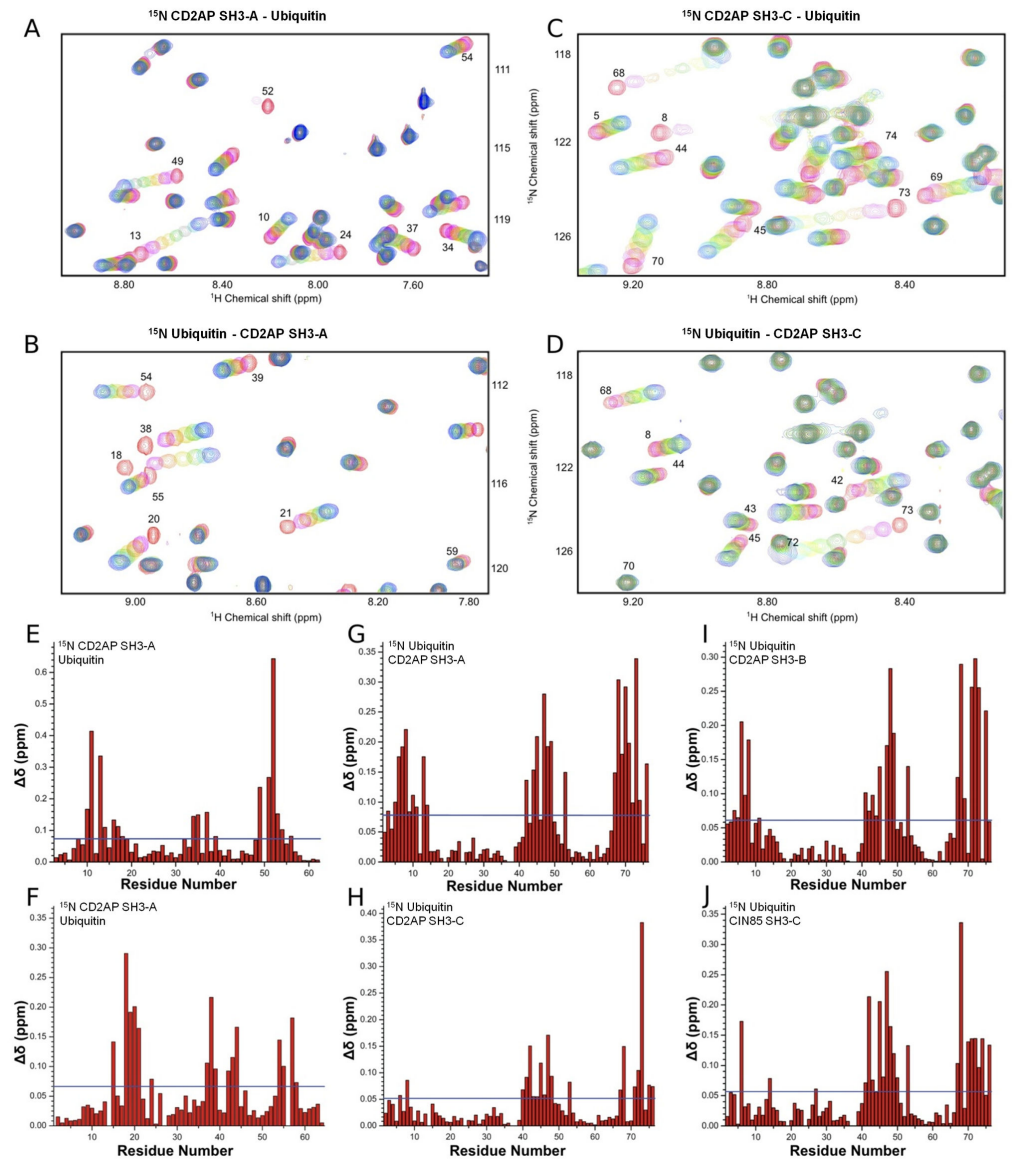
Monitoring the binding between ubiquitin and the CD2AP SH3 domains and the CIN85 SH3-C domain by NMR chemical shift perturbations. **a**. ^1^H and ^15^N chemical shift changes upon titrating ubiquitin into a ^15^N-labelled CD2AP SH3-A solution. Region of ^1^H-^15^N HSQC spectra recorded at increasing amounts of ubiquitin up to a final ratio of 1:1.6 (SH3: Ubi) (red to blue). **b**. ^1^H and ^15^N chemical shift changes upon titrating ubiquitin into a ^15^N-labelled CD2AP SH3-C solution. Region of ^1^H-^15^N HSQC spectra recorded at increasing amounts of ubiquitin up to a ratio of 1:2.4 (SH3: Ubi) (red to blue). **c**. ^1^H and ^15^N chemical shift changes upon titrating CD2AP SH3-A into a ^15^N-labelled ubiquitin solution. Region of ^1^H-^15^N HSQC spectra recorded at increasing amounts of CD2AP SH3-A up to a ratio of 1:1.4 (Ubi: SH3) (red to blue). **d**. ^1^H and ^15^N chemical shift changes upon titrating CD2AP SH3-C into a ^15^N-labelled ubiquitin solution. Region of ^1^H-^15^N HSQC spectra recorded at increasing amounts of CD2AP SH3-C up to a ratio of 1:2.5 (Ubi: SH3) (red to blue). **e** & **f**. Chemical shift deviations (Δδ) between the first and last titration point of the ubiquitin titration into ^15^N-labelled CD2AP SH3-A and SH3-C, respectively. **g**, **h**, **i** & **j**. Chemical shift deviations (Δδ) between the first and last titration point of titrating CD2AP SH3-A (G), SH3-C (H), SH3-B (I) and CIN85 SH3-C (J) into ^15^N-labelled ubiquitin corresponding to a ratio of 1:1.4, 1:2.5, 1:1.2 and 1:1.1 (Ubi: SH3) respectively. Blue solid lines indicate the mean chemical shift deviations.

NMR chemical shifts of protein residues are highly sensitive to changes in the local environment, and chemical shift perturbations are therefore widely used to map intermolecular interfaces of protein complexes [31]. Titrations of ^15^N-labeled ubiquitin with increasing amounts of unlabeled SH3-A and C domains at 25°C and vice versa caused a selective shift of both amide proton and nitrogen resonances of several ubiquitin, SH3-A (Figure 1a and c) and SH3-C residues (Figure 1b and d), indicating a specific interaction between the two proteins. The most significant changes in the chemical shift of the HSQC cross-peaks of the SH3-A domain are observed at the RT loop (residues 10-11, 13-14 and 16-18), the n-Src loop (residues 34-35), at the beginning of the β-III strand (residues 37 and 39) and at the 3^10^ helix and β-V strand (residues 49-56) (Figure 1e). While the titration profiles for SH3-A show intermediate exchange behaviour, fast exchange is observed in the case of SH3-C, indicating less tight binding for the third CD2AP SH3 domain. Substantial chemical shift differences between the free and bound form of SH3-C are found within the RT (residues 283-288) and the n-Src (residues 302-304) loops, the beginning of the βIII -strand (residues 307-309) and in residues 319-323 located at the beginning of the 3^10^ helix (Figure 1f). Different chemical shift perturbation profiles are also found when titrating the two domains into ^15^N-labelled ubiquitin. Titration with unlabelled SH3-A causes considerable changes in the chemical shifts of residues in mainly three regions of the protein, around Leu8 and Ile44, a common binding region for most UBDs, and around Val70 (Figure 1g). These three regions are affected in a similar extent. In contrast, titration of ^15^N ubiquitin with unlabelled SH3-C induces most significant changes in the Ile44 and Gly76 binding regions of ubiquitin, and very small changes are observed around Leu8 (Figure 1h).

In the light of these different results obtained for the two SH3 domains of CD2AP (A and C), we decided to investigate a possible interaction between the second domain (SH3-B) and ubiquitin. Chemical shift perturbations observed when titrating ^15^N-labeled ubiquitin with CD2AP SH3-B are very similar to that of CD2AP SH3-A (Figure 1i), suggesting an analogous ubiquitin binding mode for these two N-terminal SH3 domains of CD2AP.

### The three dimensional structure of the complex between the CD2AP SH3-A and C domains with ubiquitin

Despite the high sequence homology between the three SH3 domains of CD2AP, the different chemical shift perturbations patterns indicate structural differences between the SH3: Ubiquitin complexes. We have determined the structures of the complexes CD2AP SH3-A and SH3-C in interaction with ubiquitin in order to understand the basis of these differences and to compare with previously determined structures of SH3: Ubiquitin complexes (PDB entries 2JT4 and 2K6D). All backbone resonances of the complex forms of SH3-A and C and ubiquitin were assigned by following the cross-peaks of the free forms upon titration with the partners and subsequently checked using 3D HNCACB, CBCA(CO)NH and HBHA(CO)NH experiments.

#### Solution structure of the SH3-A and SH3-C:Ubiquitin complexes

**Figure 2:**
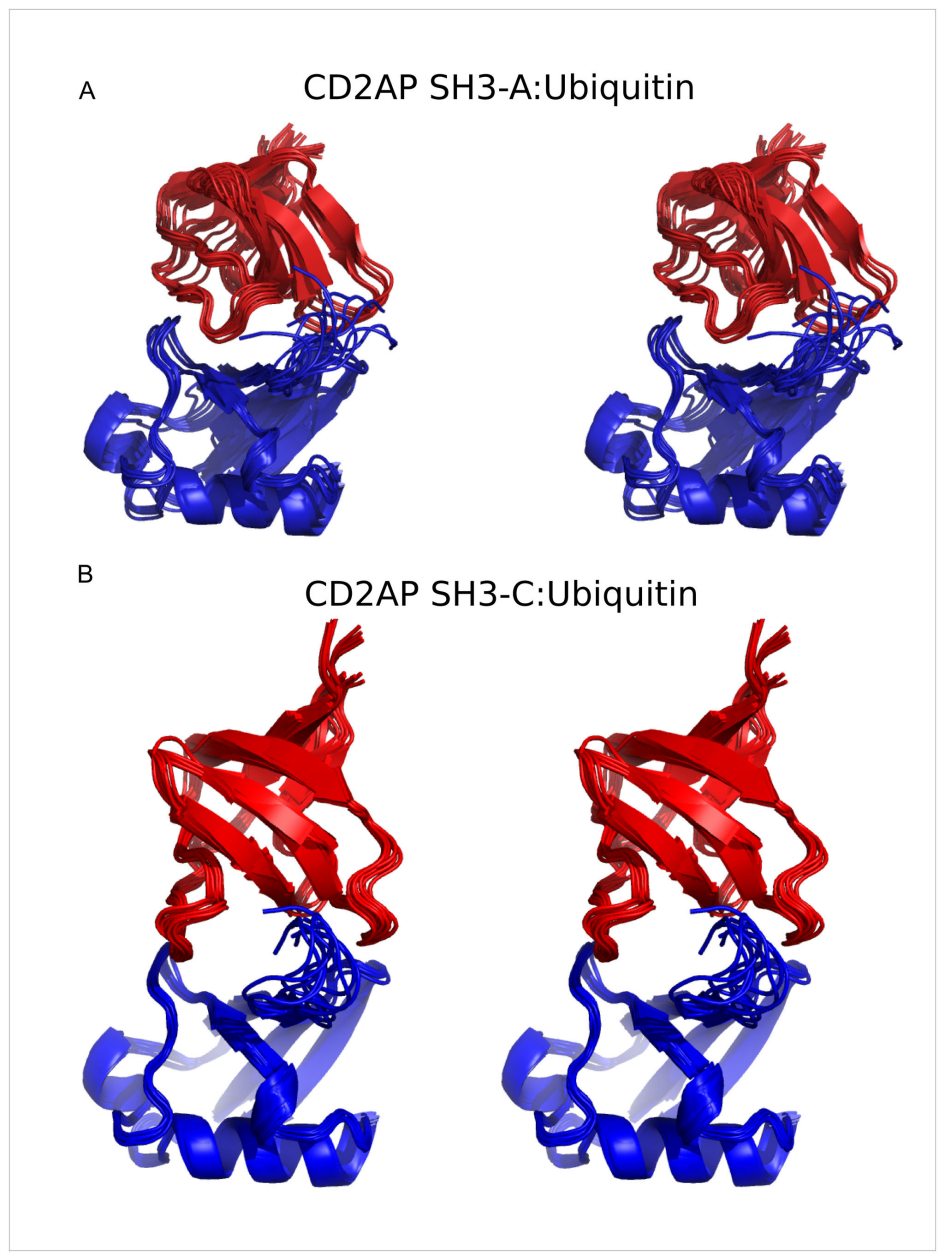
Solution structures of the complexes between ubiquitin and the first and third SH3 domains of CD2AP. Stereo representation of the ensemble of 10 lowest combined target function structures of the CD2AP SH3-A (**a**) and SH3-C (**b**) domains in complex with ubiquitin. Ubiquitin is represented in blue and the SH3 domain in red. Structures were superimposed on the backbone atoms of residues 7-70 in ubiquitin.

We have recently described a novel approach for the study of weak macromolecular complexes [15] that was applied to the SH3-C:Ubiquitin complex. Figure 2b shows a stereo representation of the ensemble of the lowest target function structures (combining RDC, AIR and nOe violation) of CD2AP SH3-C in complex with ubiquitin. The ensemble shows very good Ramachandran statistics while fulfilling the experimental data (Table S1), with an average backbone RMSD for residues 8-62 in SH3-C and 6-70 in ubiquitin to the mean of 0.5 ± 0.1 Å.

A similar approach has been applied to elucidate the SH3-A:Ubiquitin complex structure. The stereo representation of the ensemble of the lowest target function structures (combining RDC and AIR violations) of CD2AP SH3-A in complex with ubiquitin is shown in Figure 2a. Structural statistics are summarized in Table S1. The average backbone RMSD for residues 3-56 in SH3-A and 6-70 in ubiquitin to the mean is 0.9 ± 0.2 Å.

#### Comparison between the different SH3: Ubiquitin complexes

In contrast to the SH3-C:Ubiquitin complex, the structure of the SH3-A:Ubiquitin complex has a comparable orientation to the previously reported Sla1 SH3-3:Ubiquitin structure (PDB entry 2JT4). The total surface area that becomes buried upon complex formation is 1186 and 1166 Å2 for CD2AP SH3-A and C complexes, respectively, in comparison to 1197 Å2 for the Sla1 SH3-3 complex.

**Figure 3:**
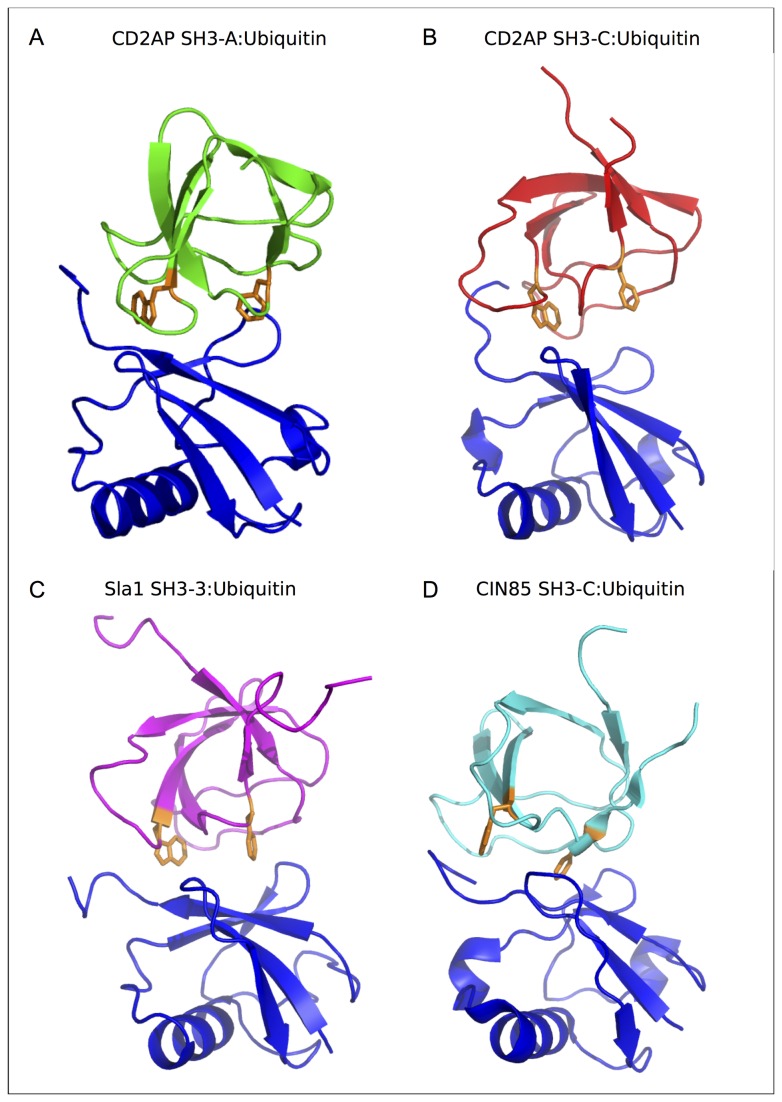
Structural comparison of different SH3: Ubiquitin complexes. Cartoon representation of the lowest target function or lowest energy structure of different SH3: Ubiquitin complexes. **a**. CD2AP SH3-A:Ubiquitin complex. Ubiquitin is represented in blue and CD2AP SH3-A in green. **b**. CD2AP SH3-C:Ubiquitin complex. Ubiquitin is represented in blue and CD2AP SH3-C in red. **c**. Sla1 SH3-3 in complex with ubiquitin (PDB entry 2JT4; ubiquitin in blue, Sla1 SH3-3 in magenta). **d**. CIN85 SH3-C in complex with ubiquitin (PDB entry 2K6D; ubiquitin in blue, CIN85 SH3-C in cyan). Phenylalanine and tryptophan residues equivalent to F53 and W41 in CD2AP SH3-A are given in stick representation and coloured in orange in all SH3 domains for clarity. All complexes were superimposed on the backbone atoms of residues 7-70 of ubiquitin.

Both SH3 domains bind to the same interface of ubiquitin, which involves the Ile/Leu-rich patch, the region of the protein that is most commonly found in complex with UBDs [9,10], but differs mainly in the type of residues surrounding this hydrophobic patch. Both complexes target the Ile44 binding region on ubiquitin in a similar way via the 310 helix, including Phe53 (Phe409 in Sla1 SH3-3) and Trp37 (Trp391 in Sla1 SH3-3). CD2AP SH3-A (Figure 3a) domain adopts a similar orientation to that of Sla1 SH3-3 in its complex with ubiquitin although with a small displacement towards the Leu8 ubiquitin binding region (Figure 3d) due to contacts with the CD2AP SH3-A n-Src loop, whereas this loop in Sla1 SH3-3 engages the Val70 ubiquitin binding region. This displacement in the CD2AP SH3-A:Ubiquitin complex is in agreement with the large number of residues showing chemical shift perturbations (CSP) in the Leu8 region of the ubiquitin molecule (Figure 1g) and those found for the charged residues in the n-Src loop, suggesting the formation of intermolecular interactions (Figure 1e). The RT loop targets different areas in both complexes. While in the Sla1 SH3-3 complex it lies at the edge of the Ile44 binding region, in the CD2AP SH3-A complex the RT loop engages the first residues in the ubiquitin C-terminus, in agreement with the large CSP found in both regions.

The binding interface for CD2AP SH3-C (Figure 3b) engages both the Ile/Leu-rich hydrophobic patch and the C-terminal diglycine patch on ubiquitin but in a different way to that for CD2AP SH3-A and Sla1 SH3-3 domains described above. This orientation is dominated by the proximity the n-Src loop to Arg74 and the diglycine 75-76 patch in ubiquitin consistent with the high chemical shift perturbation observed in the C-terminus of ubiquitin (Figure 1h). Furthermore, the Gly76 binding region is targeted as well, although at a lower extent, through the RT loop, as well as the Ile44 binding. Trp308 lies in the proximity of Val70 and Leu8 areas in ubiquitin, as well as the 310 helix, but Phe324 is not involved in any contact with ubiquitin.

### Mutational Analysis of the CD2AP SH3: Ubiquitin interactions

**Figure 4:**
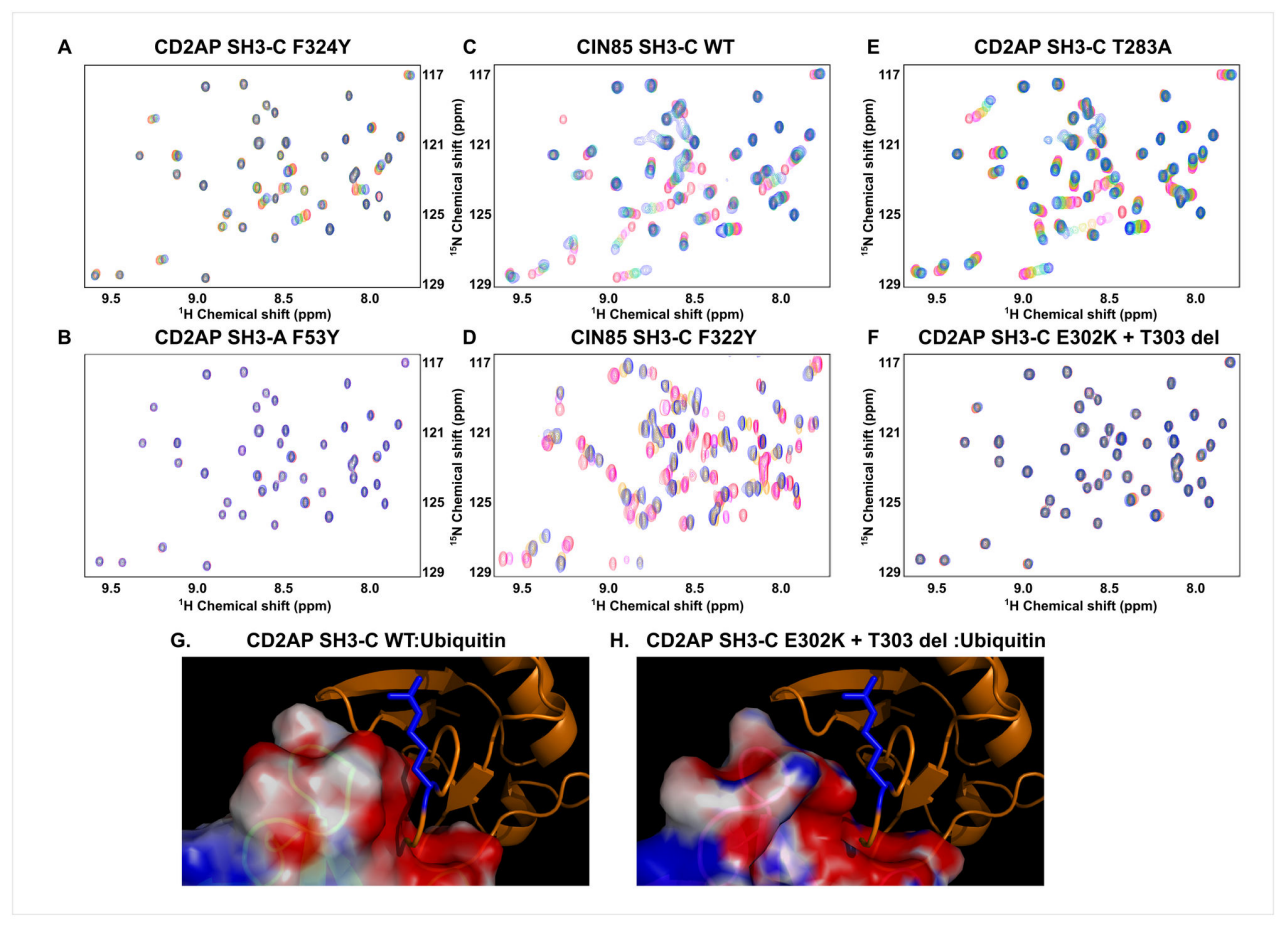
Mutational study of the ubiquitin binding by the CD2AP SH3-A and C domains and the CIN85 SH3-C domain. Region of ^1^H-^15^N HSQC spectra recorded at increasing amounts of the CD2AP or CIN85 SH3 mutant (red to blue). **a**. ^1^H and ^15^N chemical shift changes upon titrating the CD2AP SH3-C F324Y mutant into a ^15^N-labelled ubiquitin sample up to a ratio of 1:0.9 (Ubi: SH3). **b**. ^1^H and ^15^N chemical shift changes upon titrating the CD2AP SH3-A F53Y mutant into a ^15^N-labelled ubiquitin sample up to a ratio of 1:1 (Ubi: SH3). **c**. ^1^H and ^15^N chemical shift changes upon titrating the CIN85 SH3-C wild type form into a ^15^N-labelled ubiquitin sample up to a ratio of 1:1 (Ubi: SH3). **d**. ^1^H and ^15^N chemical shift changes upon titrating the CIN85 SH3-C F322Y mutant into a ^15^N-labelled ubiquitin sample up to a ratio of 1:1.2 (Ubi: SH3). **e**. ^1^H and ^15^N chemical shift changes upon titrating the CD2AP SH3-C T283A mutant into a ^15^N-labelled ubiquitin sample up to a ratio of 1:1.4 (Ubi: SH3). **f**. ^1^H and ^15^N chemical shift changes upon titrating the CD2AP SH3-C E302K + T303 deletion mutant into a ^15^N-labelled ubiquitin sample up to a ratio of 1:1.1 (Ubi: SH3). **g** and **h**. Electrostatic surface representation of the interaction between the ubiquitin C-terminus and the n-Src loop of the WT CD2AP SH3-C and the CD2AP SH3-C E302 + T303 deletion mutant respectively. Ubiquitin R74 side chain is represented in blue sticks for clarity.

Ubiquitin-binding activity by SH3 domains has been linked to the presence of a phenylalanine in equivalent positions to F409 in the Sla1 SH3-3 domain [11]. Close inspection of both CD2AP SH3: Ubiquitin complexes indicate that phenylalanine 53 in CD2AP SH3-A (Figure 3a) is located at the core of the hydrophobic interface, in a similar position to that occupied by the equivalent phenylalanine in both the CIN85 SH3-C and the Sla1 SH3-C:ubiquitin complexes (Figure 3c and 3d; PDB 2K6D and 2JT4). In contrast, phenylalanine 324 in CD2AP SH3-C (Figure 3b) is not directly involved in binding as it is placed at the edge of the binding interface. Thus, mutation of this phenylalanine to a tyrosine in the CD2AP SH3-C domain is not expected to abolish ubiquitin binding as opposed to what was observed for Sla1 SH3-3. Indeed ^1^H-^15^N HSQC spectra recorded on a ^15^N-labeled ubiquitin sample with increasing amounts of the SH3-C F324Y mutant (Figure 4a) indicate that the mutant binds ubiquitin in a similar way to that observed for the WT protein. Equivalent experiments were performed with CD2AP SH3-A F53Y mutant. In this case though, the F53Y mutation in CD2AP SH3-A abolished binding as observed from the ^1^H-^15^N HSQC spectra (Figure 4b), in agreement with our structural model and with the mutational analysis performed on Sla1 SH3-3 [11].

To confirm our NMR studies we performed fluorescence and Isothermal Titration Calorimetry (ITC) experiments on WT and the F324Y mutant of CD2AP SH3-C with ubiquitin (Figure S1). Both SH3-C variants bind ubiquitin with a moderate but similar affinity with a K_d_ of 12 ± 2 µM and 24 ± 3 µM for the WT and the F324Y mutant, respectively. WT CD2AP SH3-A binds ubiquitin with substantially higher affinity, K_d_ of 1.9 ± 0.3 µM (Figure S1).

### Effect of the extension of the ubiquitin *C-*terminus in SH3 binding

**Figure 5:**
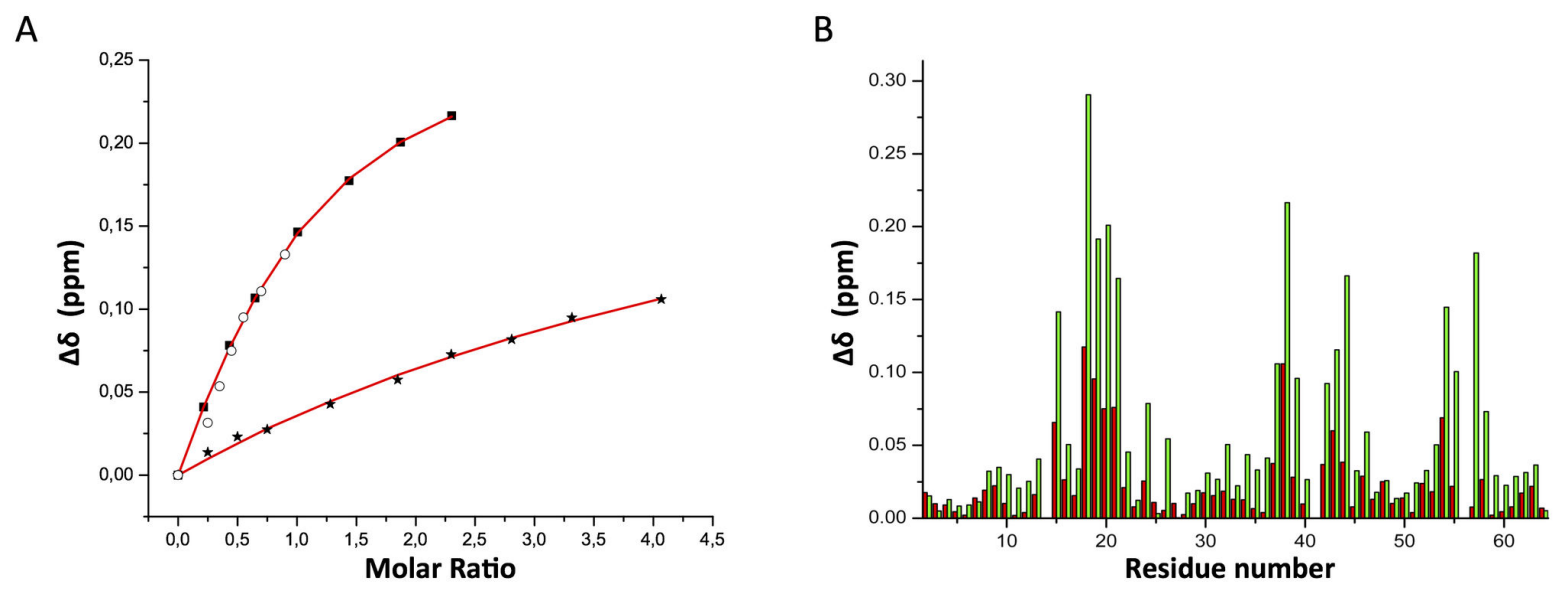
The effect of a C-terminal tag in ubiquitin on SH3 binding. **a**. Titration curve monitored by NMR chemical shift perturbations of T283 (black) and E303 (red) in CD2AP SH3-C. The upper curves correspond to titrations with C-terminal His-tagged ubiquitin, while the lower curves correspond to titrations with non-tagged ubiquitin. **b**. Comparison of the chemical shift deviations (Δδ) between the first and last titration point of titrating ^15^N-labelled CD2AP SH3-C with C-terminal His-tagged ubiquitin (green) and non-tagged ubiquitin (red).

Hicke and co-workers showed that some of the ubiquitin interacting SH3 domains bind significantly better to ubiquitin when GST was covalently attached to its C-terminus, including the CIN85 SH3-C domain and the amphiphysin SH3 domains [11]. We investigated whether such behaviour is also found for the CD2AP SH3-A and SH3-C domains and whether this is somehow related to the difference observed in the ubiquitin-binding mode. Figure 5a shows NMR-detected titrations of non-His tagged ubiquitin versus C-terminal His-tagged ubiquitin into ^15^N-labelled SH3-C. Although no chemical shift difference of the His-tag is observed in the 1H-15N HSQC between the free and bound forms and no differences are found in the ubiquitin areas targeted by the SH3 domain (Figure 5b), one order of magnitude higher K_d_ values are obtained for the titration of SH3-C domain with non His-tagged ubiquitin. In contrast, such a significant difference in affinity is not observed in ubiquitin binding by the CD2AP SH3-A domain, by the highly homologous CIN85 SH3-A (68% identity) and by Sla1 SH3-3 [11]. In light of these results, and due to the higher CIN85 SH3-C affinity to C-terminal GST-tagged ubiquitin reported before [11], a NMR detected titration was carried out with unlabelled CIN85 SH3-C and ^15^N-labelled C-terminal His-tagged ubiquitin (Figure 4c). Interestingly, our NMR titrations suggest significantly tighter binding as well as different areas affected in ubiquitin upon binding compared to those previously reported by Forman-Kay and co-workers using non-tagged ubiquitin [12] (Figure 1). Furthermore, the ubiquitin areas targeted in this work are very similar to the CD2AP SH3-C titration. According to these results and the high sequence identity (53.3%) between the two SH3-C domains, we produced the CIN85 F322Y SH3-C mutant and titrated increasing amounts of it into ^15^N labelled ubiquitin. Although it was stated that this mutant abolishes ubiquitin binding due to the replacement of the key Phe322 residue as in Sla1 SH3-3 [12], we observed binding with a similar affinity to that observed for the wild type form (Figure 4d), indicating that CIN85 SH3-C displays a comparable behaviour to CD2AP SH3-C towards ubiquitin binding.

### High resolution X-ray structure of the free CIN85 SH3-C domain

As we indicated before, the mutation of Phe322 to a tyrosine in the CIN85 SH3-C domain does not abolish ubiquitin binding. However, in the structure of this complex (PDB 2K6D, Figure 3c) this Phe is located at the heart of the binding interface. As the CIN85 SH3-C:Ubiquitin complex was calculated starting from a homology model of the mouse CIN85 SH3-C domain (PDB 2DA9), which might have biased the structure determination of the complex, we have solved the high resolution structure of CIN85 SH3-C domain using X-ray crystallography at a resolution of 2.05 Å (Figure S2a and b). The determined structure is very similar to the recently published NMR structure ( [32], PDB 2K9G), with a backbone RMSD of 0.623 Å, but significantly different to the CIN85 SH3-C in complex with ubiquitin (PDB 2K6D), with a backbone RMSD of 1.307 Å (Figure S2c).

## Discussion

Ubiquitin is known to regulate a wide variety of cellular activities ranging from transcriptional regulation to cell signalling and membrane trafficking. Many cellular activities of ubiquitin are known to be mediated by mono- rather than poly-ubiquitin, and its functions are deciphered by various ubiquitin-binding proteins. Recently, it was found that SH3 domains constitute a new, distinct type of ubiquitin-binding domains [11]. The structure of the complex between Sla1 SH3-3 and ubiquitin shows that the ubiquitin binding surface largely overlaps with the canonical binding surface for proline-rich ligands and as in many other ubiquitin-binding motifs, the SH3 domain engages the Ile44 hydrophobic patch of ubiquitin [13]. A key affinity and specificity determinant for ubiquitin-binding was appointed to be Phe409 of Sla1, located at the heart of the hydrophobic interface in the SH3-ubiquitin complex, and a tyrosine residue at that position was found to abrogate ubiquitin binding.

We previously described a new method to use NMR RDCs to obtain the solution structure of the complex between ubiquitin and the SH3-C domain of CD2AP [15]. In the current work, we have also used RDCs to obtain the solution structure of the complex between ubiquitin and the first (SH3-A) domain of CD2AP. In this domain, the mutation of Phe53 (equivalent to Phe409 in Sla1 SH3-3) to tyrosine abolishes ubiquitin binding similarly to what was observed for Sla1 SH3-3. In contrast, the mutation of the corresponding phenylalanine residue in CD2AP SH3-C only has a minor effect on ubiquitin binding as observed in both the NMR and ITC titration experiments. The results obtained here demonstrate that the two SH3 domains from the same adaptor protein show alternative modes of ubiquitin binding. The intriguing question remaining is which residues and/or what region of a particular ubiquitin-binding SH3 domain determine the mode and the orientation of binding to ubiquitin and furthermore, whether this difference in binding mode is related to a difference in function.

**Figure 6:**
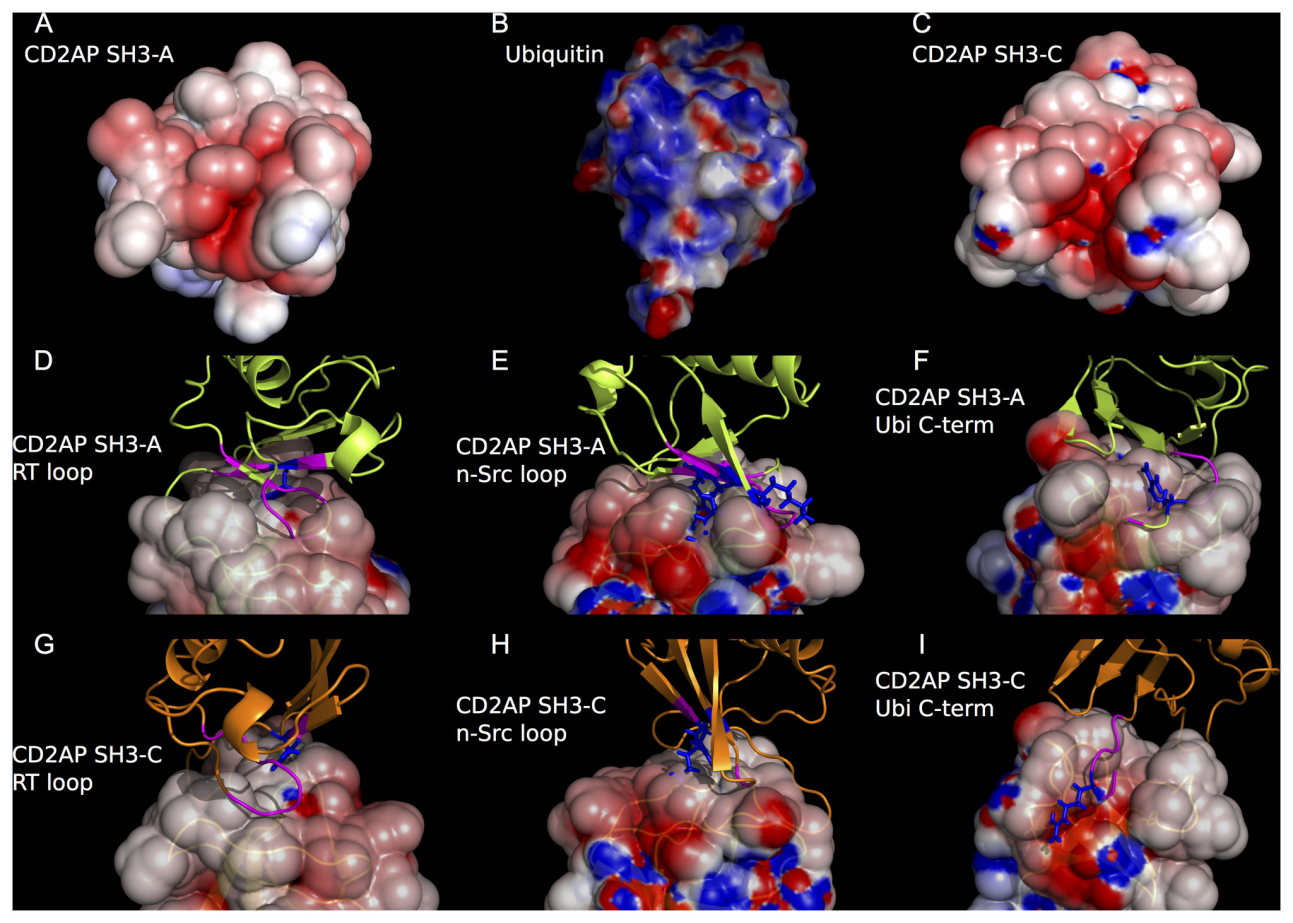
SH3: Ubiquitin binding interfaces. Surface representations of the free forms of CD2AP SH3-A (**a**), ubiquitin (**b**) and CD2AP SH3-C (**c**), showing the surface-charge distribution. Electrostatic surface representation of the orientation of the CD2AP SH3-A and C complexes with ubiquitin (green and orange respectively), zooming in at the SH3 RT-loop (**d** & **g** respectively), SH3 n-Src loop (**e** & **h** respectively) and ubiquitin C-terminus (**f** & **i** respectively). Ubiquitin residues with chemical shift perturbation higher than the mean are coloured in magenta. Charged residues with high CSP are represented in coloured sticks (blue and red for positively and negatively charged) for clarity. The electrostatic surface representation was drawn with Pymol (www.pymol.org) using a Poisson-Boltzmann electrostatics calculation [40].

The distinct interactions between SH3 domains and ubiquitin can be rationalized by analyzing the surfaces of both proteins. Figure 6c shows that the CD2AP SH3-C binding surface is mainly negatively charged, with a shallow and highly negatively charged groove between the RT and n-Src loops, in contrast with the less polar and smoother surface at the complex interface in CD2AP SH3-A (Figure 6a) and Sla1 SH3-3. A close inspection of the disposition of the different SH3: Ubiquitin structures reveals that the main differences between both binding modes reside in the interactions between polar residues in the RT and n-Src loops and the ubiquitin C-terminus.

Comparison of the amino acid sequences of CD2AP SH3-A and C, Sla1 SH3-3 and CIN85 SH3-C domains (Figure S3) shows that the residues found in the RT loop are generally alike, except for the presence of two polar residues at positions 281 (Thr) and 283 (Thr) in CD2AP SH3-C that correspond to a negatively charged and a hydrophobic residue respectively in CD2AP SH3-A and Sla1 SH3-3 domains. In both cases the RT loop targets the edge of the Ile44 binding region in ubiquitin, although in the CD2AP SH3-A domain it also targets the first residues of the ubiquitin C-terminus with the n-Src loop. In fact, and despite the high flexibility of the ubiquitin C-terminus, a close disposition of both is found in all the structures of the ensemble (Figures 2A and 6). Bigger differences are observed within the n-Src loop. This loop in CD2AP and CIN85 SH3-C is one residue longer, resulting in a different position of the sidechains and creating a shallow and highly negatively charged groove with the RT loop, accommodating the ubiquitin C-terminus on its surface. The disposition of the CD2AP SH3-C n-Src amino acid sidechains towards the groove results as well in fewer contacts to the Leu8 ubiquitin binding region, that are only possible by shifting this region towards the n-Src loop. Finally, this causes a big structural rearrangement of the 3^10^ helix with respect to the free CD2AP SH3-C structure, all resulting in Phe324 being outside the binding interface. In contrast, the orientation of the CD2AP SH3-A domain n-Src loop permits a close disposition to the Leu8 binding region in ubiquitin, shifting the complex interface towards this area of ubiquitin when compared with the CD2AP SH3-C and Sla1 SH3-3 complexes.

To rationalize the importance of the n-Src loop over the RT loop in the CD2AP SH3-C:Ubiquitin interaction, we constructed two CD2AP SH3-C mutants, one in which Thr283 in the RT loop was mutated to alanine, and one in which Glu302 in the n-Src loop was mutated into a lysine together with the deletion of Thr303. The latter mutant thus becomes more similar to the Sla1 SH3-3 domain in terms of charge and length of the n-Src loop. No significant differences in ubiquitin binding were found between the WT and the T283A mutant (Figure 4e), similarly to what was previously reported for the glutamate to alanine mutation at the equivalent position in Sla1 SH3-3 [11]. In contrast, the E302K + T303 deletion mutant completely eliminates ubiquitin binding (Figure 4f), thus pointing to the n-Src loop as the key region responsible for this ubiquitin binding mode. To discard that deficiency of ubiquitin binding resulted from an unfolded SH3-C domain due to the mutation, 1D ^1^H experiments were recorded throughout the NMR titration experiments. These spectra indicate proper folding of the SH3-C mutant.

We constructed a structural model of the CD2AP SH3-C E302K + T303 deletion mutant using the program Modeller [33]. The shorter n-Src loop pushes this region further away from the ubiquitin C-terminus (Figure 4g and h). Due to the shorter length of the loop, the replaced Glu302 to a Lys points outwards of the groove, thus eliminating any hydrophobic interaction with Gly75 and hindering the interaction of Gly304 with the Arg74 in ubiquitin. Moreover, the rearrangement introduces some positively charged areas in the binding interface that result in unfavourable contacts with Arg74 in ubiquitin. As stated before, positively charged and non-polar residues compose the same regions in CD2AP SH3-A and in Sla1 SH3-3. These residues are not located in the proximity of the binding interface. Both differences in length and charge of the n-Src loop are thus related to the different ubiquitin binding modes exhibited by SH3 domains. All mutations performed in ubiquitin binding SH3 domains and their effects are summarized in Figure S3.

Hicke and co-workers showed that some of the ubiquitin interacting SH3 domains bind significantly better to ubiquitin when GST was covalently attached to its C-terminus, including the CIN85 SH3-C domain and the amphiphysin SH3 domains [11]. The importance of the ubiquitin C-terminus is not novel in SH3: Ubiquitin binding as observed in the interaction between Endophilin-A SH3 and the Parkin ubiquitin like protein (Ubl) [34] and in the interaction between NcK SH3-3 and ubiquitin [16]. Although no structure of the complex with ubiquitin was reported, the authors showed that Nck SH3-3 domain interacts with ubiquitin even though it possesses a tyrosine residue instead of a phenylalanine at the key position 73. Interestingly enough, chemical shift perturbations (CSP) suggest that the ubiquitin C-terminus is directly involved in Nck SH3 binding, although the CSP pattern found in ubiquitin and the Nck SH3-3 domain interaction is different to that reported for the Sla1 SH3-3 and that for the CD2AP SH3-C ubiquitin binding modes. In the case of the interaction between Endophilin-A SH3 and the Parkin Ubl the relative orientation is considerably different than that of Sla1 SH3-3 or CD2AP SH3-C. The Parkin Ubl C-terminus adopts an extended β strand conformation that mimics a proline-rich ligand. Moreover, Arg75 mutation to a Gly residue, as in ubiquitin, completely abolishes binding, whereas Lys76 to Gly mutation only diminishes the interaction [34]. Furthermore, the Endophilin-A SH3 domain is considerably shifted towards the C-terminus compared with the CD2AP SH3-C domain. Therefore, CD2AP SH3-C:Ubiquitin interaction constitutes a different ubiquitin binding mode than those described for Sla1 SH3-3 domain and for Endophilin-A SH3 domains.

The presence of a C-terminal tag in ubiquitin has a strong effect on the ubiquitin affinity for CD2AP SH3-C and CIN85 SH3-C but not for CD2AP SH3-A domain. We previously showed a marked decrease in the flexibility of the ubiquitin C-terminus in the SH3-C complex using spin relaxation data [35]. The C-terminal extension results in rigidification of the ubiquitin C-terminus, lowering the entropy cost associated with the decrease of its flexibility upon binding, as recently observed for the BAR SH3-Ubl interaction [34]. The changes in internal dynamics in the free ubiquitin molecules would therefore be reflected in favourable changes in conformational entropy, and thus an increase in binding affinity, as it has been shown for the catabolite activator protein-DNA binding [36]. The higher affinity to C-terminal elongated ubiquitin molecules was previously reported for CIN85 SH3-C and amphiphysin SH3 domains binding to ubiquitin [11], although in this case the His-tag was replaced by a GST-tag. These domains, like CD2AP SH3-C, posses a longer n-Src loop, as well as negatively charged residues at the edge of it, suggesting that they bind ubiquitin in a similar manner to that of CD2AP SH3-C when binding C-terminal tagged ubiquitin. This suggests that the higher affinity observed for C-terminal tagged ubiquitin constitutes a feature of this binding mode.

Titrations of the highly homologous (53.3% identity) CIN85 SH3-C into ^15^N-labelled C-terminal His-tagged ubiquitin (Figure 1j) revealed a CSP pattern very similar to the one exhibited by CD2AP SH3-C (Figure 1f). Furthermore, the F322Y mutation in the CIN85 SH3-C domain does not diminish ubiquitin binding (Figure 4c and d), and taking also into account the higher affinity of CIN85 SH3-C towards C-terminal tagged ubiquitin molecules, we can conclude that CIN85 and CD2AP SH3-C domains present a similar ubiquitin binding mode. These results are in contrast to the relative orientation of the CIN85 SH3-C domain and ubiquitin in the previously determined complex (Figure 3d [12]); the difference might be the result of a distortion due to the G76C mutation and the incorporation of a MTSL molecule into the ubiquitin C-terminus, as well as due to PRE constraints sensitive to minor alternate conformations [15]. This incorrect orientation might also be caused by the use of a structural model of the free CIN85 SH3-C domain, which was based on the mouse CIN85 SH3-C domain (PDB 2DA9) We, therefore solved the crystal structure of CIN85 SH3-C and show that this X-ray structure as well as the recently published NMR structure (PDB 2KG9 [17]) are substantially different to the same domain in complex with ubiquitin (Figure S2c). The backbone RMSD of both NMR and X-ray structures with respect to the CIN85 SH3-C domain in the ubiquitin complex are 1.307 and 1.219 Å respectively, much higher than compared to CD2AP SH3-C, with a backbone RMSD of 0.765 Å. Importantly, differences are mainly found in the RT and n-Src loops, both crucial for the interaction with ubiquitin. Interestingly, Kay and coworkers suggested that CIN85 SH3-C presents two distinct conformations when bound to ubiquitin [14]. The first binding mode, originally named (PL)_1_, presents a lower network of contacts involving Leu8 and Ile44. In contrast, His68 and Gly47–Gln49 establish contacts with the RT loop, as well as the C-terminal region of ubiquitin, in perfect agreement with the chemical shift perturbation pattern found in CIN85 SH3-C binding to C-terminal tagged ubiquitin. Moreover, no contact involving the key Phe322 is observed, suggesting that CIN85 SH3-C is able to bind ubiquitin in both the canonical (Sla1 SH3-3) and the CD2AP SH3-C mode.

Cbl E3 ubiquitin protein ligase mediates polyubiquitination of EGFR and monoubiquitination of CIN85 to control endosomal sorting and degradation of receptor tyrosine kinases. CD2AP and CIN85 become monoubiquitinated via interaction with Cbl/Cbl-b [37] upon activation of the EGF receptor. This activation leads to phosphorylation of Cbl and subsequent binding of the SH3-A and/or SH3-B domains of CIN85/CD2AP to the proline-rich region at the C-terminal region of c-Cbl [38]. Forman-Kay and co-workers have shown that the triple Phe-to-Tyr mutant in full-length CIN85 is still able to self-ubiquitinate like the WT protein, and that even in the absence of EGF stimulation CIN85 is ubiquitinated [12]. The authors suggested that in this case, none of the three CIN85 SH3 domains is able to grab ubiquitin and hence they negatively regulate CIN85/CD2AP ubiquitination. However, we have shown in our work that CIN85 SH3-C binds ubiquitin in the same way as CD2AP SH3-C, independent if a phenylalanine or tyrosine residue is present. Therefore, even though having a tyrosine at position 322, the triple Phe-to-Tyr mutant CIN85 is still capable of binding ubiquitin via its SH3-C.

CD2AP SH3-A and B domains are able to tightly bind free ubiquitin. For CD2AP and CIN85 SH3-C domains however, tight binding requires the ubiquitin molecule be attached via its C-terminus to another protein, avoiding in this way the interaction of this domain with free ubiquitin molecules present in the cellular milieu. CD2AP/CIN85 ubiquitination takes place via a covalent bond between the ubiquitin C-terminus and the sidechain of a lysine of the intact carboxyl terminus of CD2AP/CIN85 [8,37]. This process is controlled by Cbl together with the polyubiquitination of EFGR in a trimeric complex. It has been observed for different proteins containing UBDs that their own monoubiquitination results in more favourable intramolecular interactions between this attached ubiquitin molecule and their own UBDs [39]. The ubiquitin molecule covalently attached to CD2AP/CIN85 could then be an ideal candidate for the interaction with the SH3-C domain. This possible interaction may constitute the specific mechanism suggested by Dikic and co-workers [37] that prevents further addition of ubiquitin chains to monoubiquitinated CIN85. Recognition of the CIN85-ubiquitin by SH3-C might then either protect against polyubiquitination, which would ensure degradation with EGFR, or protect against deubiquitination to prevent recycling of the CIN85 to the cytoplasmic pool. Additional experiments are required to verify this hypothesis.

It was previously established that the three CD2AP/CIN85 SH3 domains negatively regulate CD2AP/CIN85 ubiquitination [12]. However, we show that the third SH3 domain of CD2AP/CIN85 binds ubiquitin with a different mechanism. On the basis of our mutational and structural work we conclude that CD2AP and CIN85 SH3-C domain interaction with ubiquitin constitutes a new ubiquitin-binding mode involved in the regulation of the ubiquitination level of CD2AP and CIN85 adaptor proteins to ensure CD2AP/CIN85 degradation upon EFG activation, and thus changes the previously established mechanism of EGF-dependent CD2AP/CIN85 mono-ubiquitination.

### Accession Codes

Coordinates of the CD2AP SH3-A and C complexes with ubiquitin have been deposited in the PDB and chemical shifts and RDC values in the BMRB with accession numbers 2MCN and 19447 and 2LZ6 and 18737, respectively. Coordinates and structure factors of the free CIN85 SH3-C domain have been deposited with accession numbers 2YDL and r2ydlsf, respectively.

## Supporting Information

Figure S1 **K_d_ determination of the ubiquitin binding by CD2AP SH3-A and C domains.**
(DOCX)

Figure S2 **The high-resolution structure of the CIN85 SH3-C domain.**
(DOCX)

Figure S3 **Sequence comparison between ubiquitin-binding and non-binding SH3 domains.**
(DOCX)

Table S1 **Structural statistics for the 10 lowest combined target function structures of CD2AP SH3-A and C domains in complex with ubiquitin.**
(DOCX)

Table S2 **Crystallographic data collection and refinement analysis of CIN85 SH3-C.**
(DOCX)
