# Supplementary material for: Distinct Ubiquitin Binding Modes Exhibited by SH3 Domains: Molecular Determinants and Functional Implications
Source: PLoS One. 2013 Sep 11;8(9):e73018. doi: 10.1371/journal.pone.0073018 (PMC3770644; doi:10.1371/journal.pone.0073018)
Supplement: Figure S1 — Kd determination of the ubiquitin binding by CD2AP SH3-A and C domains. (DOCX) [file pone.0073018.s001.docx]

**Figure S1.** K_d_ determination of the ubiquitin binding by CD2AP SH3-A and C domains. **a.** Ubiquitin binding of CD2AP SH3-A monitored by fluorescence spectroscopy at pH 6.0 and 25°C. Experimental points are shown in solid circles and the continuous line corresponds to the best fitting of the experimental data. L_T_ corresponds to the total concentration of ubiquitin (ligand). **b.** Calorimetric titration of the WT and F59Y mutant of CD2AP with ubiquitin. Binding isotherms obtained at pH 6.0 and 25°C by consecutive injections of variable volumes (ranging between 4.0 and 18 μL) of an ubiquitin solution into the WT and F59Y mutant solution. Symbols (solid circles and solid triangles for WT and F59Y, respectively) represent the experimental data for the corrected and normalized heats produced by the binding after each injection. The continuous lines (red and blue lines for WT and F59Y, respectively) correspond to the best fittings of the two isotherms (see Material and Methods). ΔQ_i_/Δ[Ubi]_T_ is the heat produced per mole of increment in the ubiquitin concentration, and [SH3]_T_ and [Ubi]_T_ are the total concentrations of CD2AP SH3-C domain and ubiquitin (ligand), respectively.

**
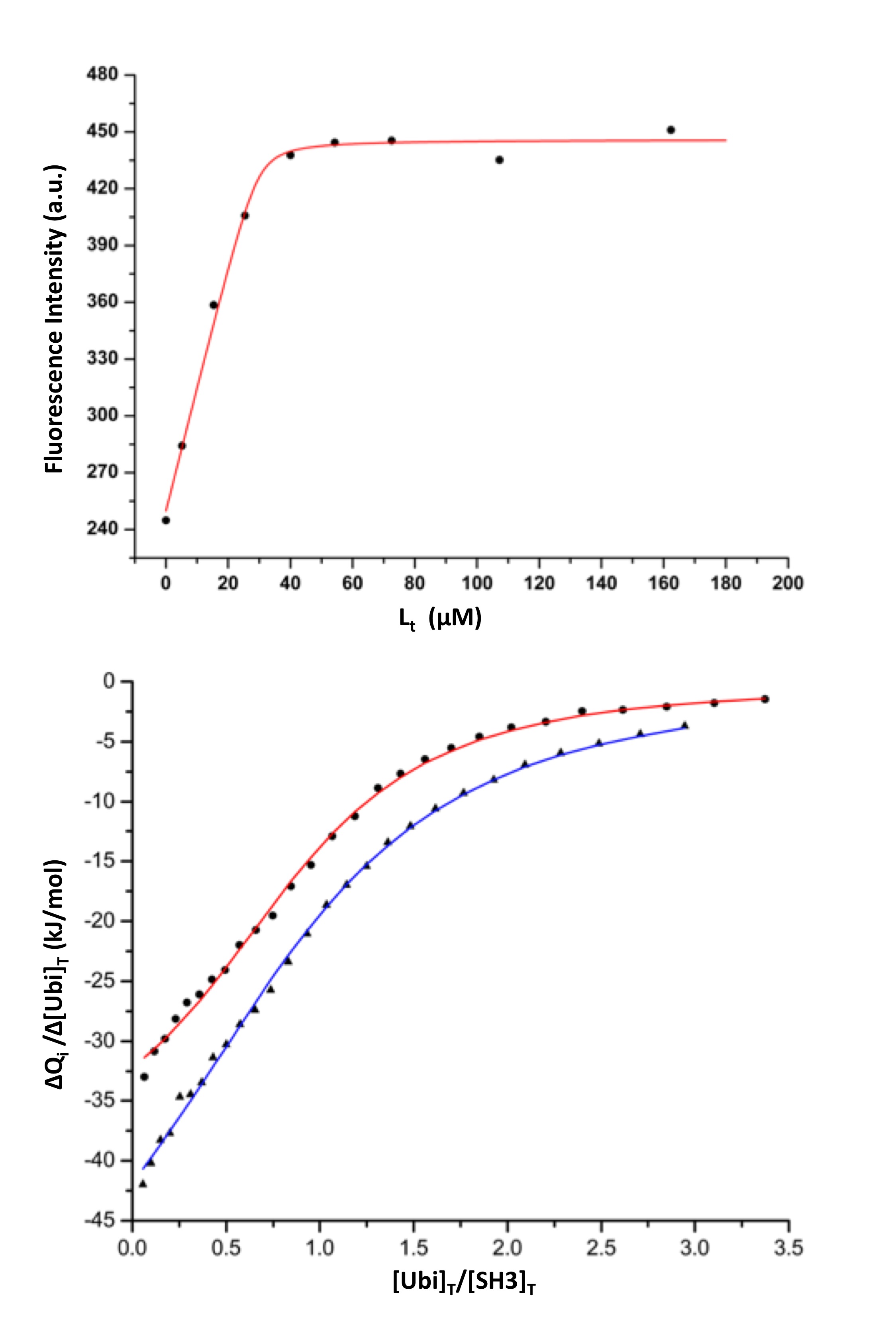
**
