# Supplementary material for: Distinct Ubiquitin Binding Modes Exhibited by SH3 Domains: Molecular Determinants and Functional Implications
Source: PLoS One. 2013 Sep 11;8(9):e73018. doi: 10.1371/journal.pone.0073018 (PMC3770644; doi:10.1371/journal.pone.0073018)

**Figure S2.** The high-resolution structure of the CIN85 SH3-C domain. **a** and **b**. The structure of the CIN85 SH3-C domain is represented in sticks. The electron density map is contoured at 2.0 σ. **c**. Structural alignment of the CIN85 SH3-C domain free (X-ray structure in green, NMR structure (PDB 2K9G) in yellow), the CIN85 SH3-C domain in complex with ubiquitin (cyan) (PDB 2K6D), and the CD2AP SH3-C domain in complex with ubiquitin (red).


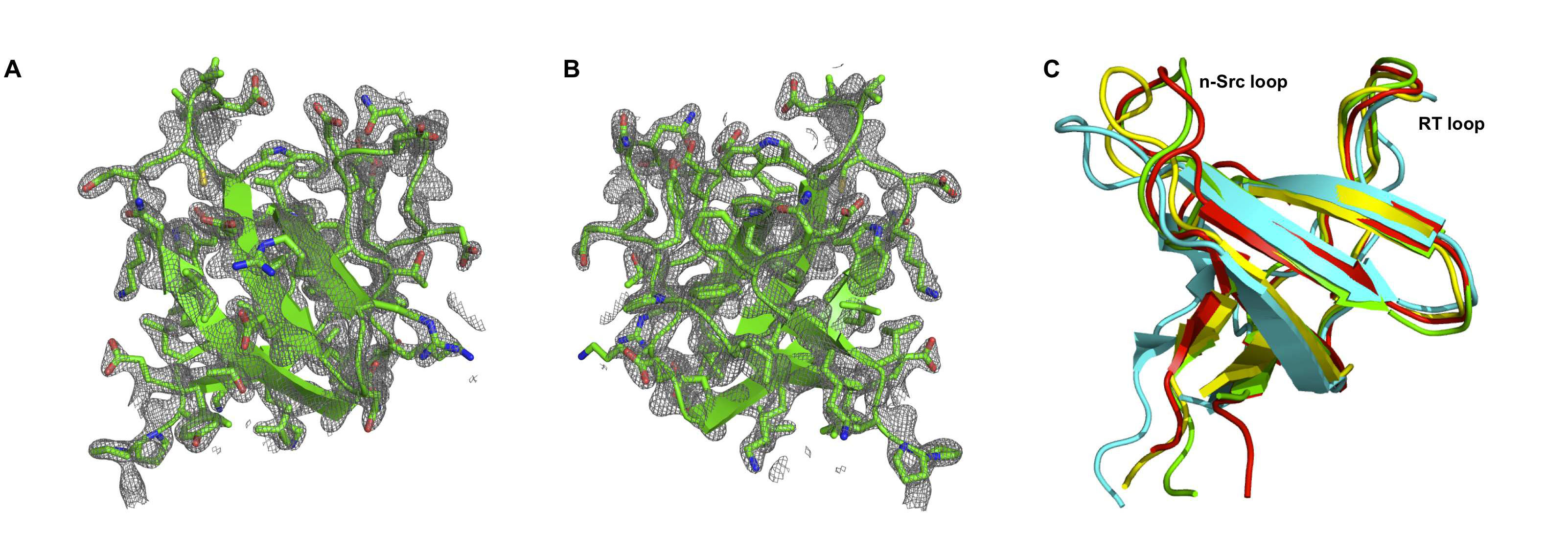

Supplement: Figure S2 — The high-resolution structure of the CIN85 SH3-C domain. (DOCX) [file pone.0073018.s002.docx]
