# Supplementary material for: Distinct Ubiquitin Binding Modes Exhibited by SH3 Domains: Molecular Determinants and Functional Implications
Source: PLoS One. 2013 Sep 11;8(9):e73018. doi: 10.1371/journal.pone.0073018 (PMC3770644; doi:10.1371/journal.pone.0073018)

**Figure S3.** Sequence comparison between ubiquitin-binding and non-binding SH3 domains. Sequence alignment of different types of SH3 domains: Group 1 is constituted by SH3 domains binding ubiquitin in a similar orientation than Sla1 SH3-3. Group 2 is constituted by SH3 domains binding ubiquitin in a similar orientation than CD2AP SH3-C. Group 3 is constituted by SH3 domains not able to bind ubiquitin. Mutations abolishing ubiquitin binding are highlighted in red, and mutations with no considerable effect are highlighted in blue ^11,12^. The black arrow points at the position of the suggested key phenylalanine residue ^11^.


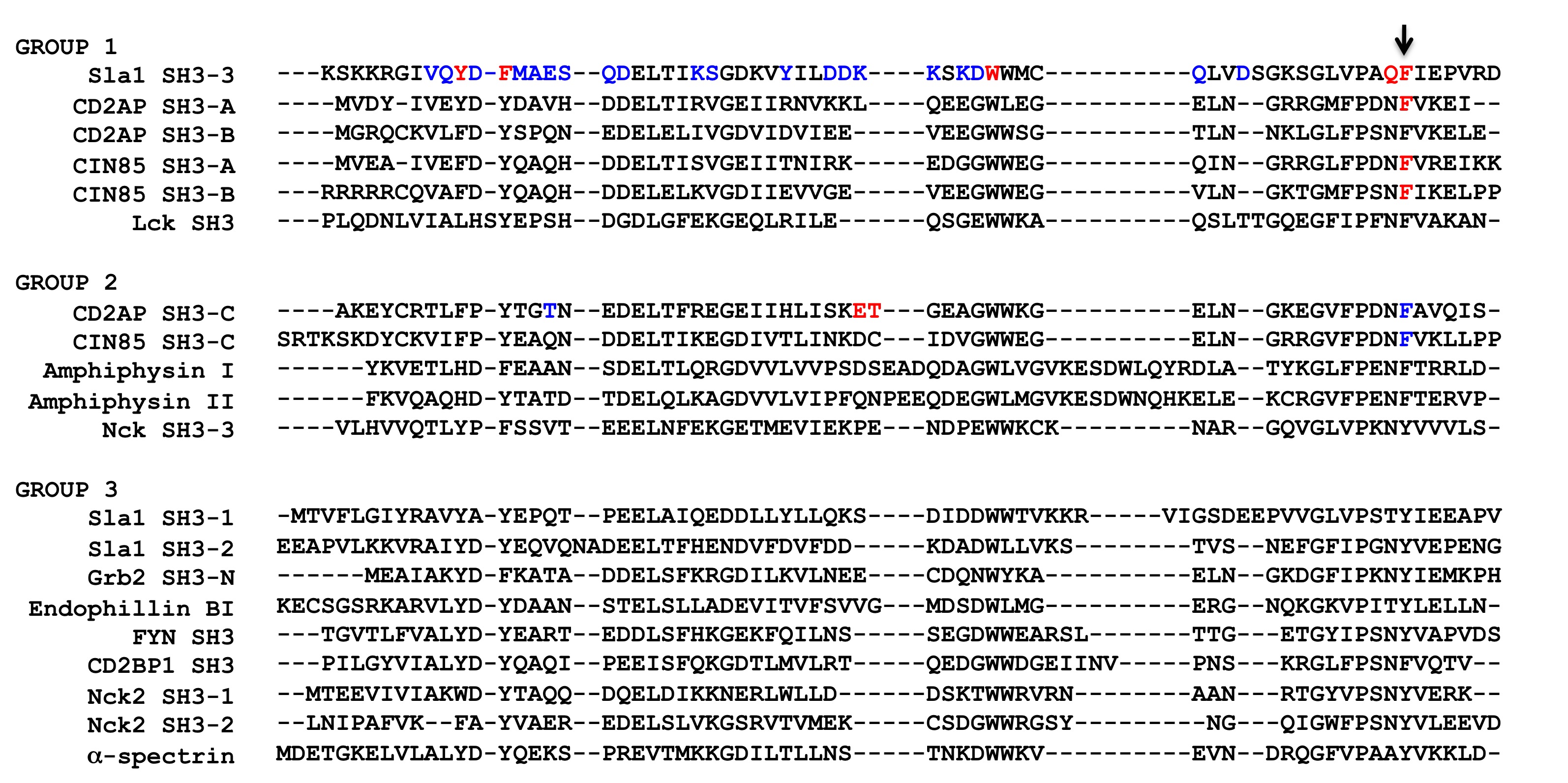

Supplement: Figure S3 — Sequence comparison between ubiquitin-binding and non-binding SH3 domains. (DOCX) [file pone.0073018.s003.docx]
