# Supplementary material for: Distinct Ubiquitin Binding Modes Exhibited by SH3 Domains: Molecular Determinants and Functional Implications
Source: PLoS One. 2013 Sep 11;8(9):e73018. doi: 10.1371/journal.pone.0073018 (PMC3770644; doi:10.1371/journal.pone.0073018)
Supplement: Table S1 — Structural statistics for the 10 lowest combined target function structures of CD2AP SH3-A and C domains in complex with ubiquitin. (DOCX) [file pone.0073018.s004.docx]

**Table S1:** Structural statistics for the 10 lowest combined target function structures of CD2AP SH3-A and C domains in complex with ubiquitin. Flexible N- and C-terminal residues were omitted from the RMSD analysis. Ramachandran statistics were obtained from PROCHECK-NMR analysis over all residues and RDC violations were calculated with MODULE ^41^.

|  | **SH3-A:Ubiquitin** | **SH3-C:Ubiquitin** |
| --- | --- | --- |
| Distance restraints |  |  |
| AIRS | 26 | 48 |
|  |  |  |
| Orientational restraints |  |  |
| Residual dipolar couplings | 107 | 227 |
|  |  |  |
| Restraint statistics |  |  |
| NOE violations > 0.5 Å | - | 0 |
| H_N_-N and C_α_-H_α_ RDC violations > 3 Hz | 0 | 7 |
| H_N_-C´ RDC violations > 1 Hz | - | 2 |
| C_α_-C´ RDC violations > 0.5 Hz | - | 3 |
|  |  |  |
| RMSD from average (Å)^1^ |  |  |
| Backbone N, CA, C´ | 0.2 ± 0.05 Å | 0.5 ± 0.1 Å |
|  |  |  |
| Ramachandran plot |  |  |
| Most favored regions (%) | 85.7 | 87.0 |
| Additional allowed regions (%) | 13.0 | 10.4 |
| Generously allowed regions (%) | 1.2 | 2.6 |
| Disallowed regions (%) | 0.0 | 0.0 |
|  |  |  |

^1^ RMSD values for residues 6-70 in ubiquitin and 3-56 in SH3-A and 8-62 in SH3-C, respectively
