# Supplementary material for: Distinct Ubiquitin Binding Modes Exhibited by SH3 Domains: Molecular Determinants and Functional Implications
Source: PLoS One. 2013 Sep 11;8(9):e73018. doi: 10.1371/journal.pone.0073018 (PMC3770644; doi:10.1371/journal.pone.0073018)
Supplement: Table S2 — Crystallographic data collection and refinement analysis of CIN85 SH3-C. (DOCX) [file pone.0073018.s005.docx]

**Table S2**. Crystallographic data collection and refinement analysis of CIN85 SH3-C.

|  | |
| --- | --- |
| Space group | P3_1_21 |
| Unit Cell Dimensions (Å) | a=b=47.83, c=70.23  α=β=90º; γ=120º |
| Data range (Å) | 41.42-2.05 |
| Observations (unique) | 19366 (5990) |
| Completeness (%) (last shell) | 96.5 (90.6) |
| R_sym_^a^ (last shell) | 0.054 (0.032) |
| Reflections F>0 (cross validation) | 5715 (275) |
| Non-hydrogen atoms (solvent molecules) | 606 (56) |
| R_cryst_^b^ (R_free_^c^) | 0.205 (0.252) |
| R.m.s. bond length (Å) | 0.016 |
| R.m.s. bond angles (^o^) | 1.419 |

^a^ R_sym_ is the unweighted R value on I between symmetry mates.

^b^ R_cryst_ = ∑_hkl_F_obs_(hkl)F_calc_(hkl)∑_hkl_F_obs_(hkl)

^c^ R_free_ is the cross-validation R factor for 4.6% of reflections against which the model was not refined.
